# LncRNA NR038975, A Serum-Based Biomarker, Promotes Gastric Tumorigenesis by Interacting With NF90/NF45 Complex

**DOI:** 10.3389/fonc.2021.721604

**Published:** 2021-11-17

**Authors:** Sisi Wei, Suli Dai, Cong Zhang, Ruinian Zhao, Zitong Zhao, Yongmei Song, Baoen Shan, Lianmei Zhao

**Affiliations:** ^1^ Research Center, The Fourth Hospital of Hebei Medical University, Shijiazhuang, China; ^2^ State Key Laboratory of Molecular Oncology, National Cancer Center/National Clinical Research Center for Cancer/Cancer Hospital, Chinese Academy of Medical Sciences and Peking Union Medical College, Beijing, China

**Keywords:** gastric cancer, lncRNA NR038975, NF90/NF45, tumorigenesis, exosome

## Abstract

Gastric cancer (GC) is one of the deadliest cancers, and long noncoding RNAs (lncRNAs) have been reported to be the important regulators during the occurrence and development of GC. The present study identified a novel and functional lncRNA in GC, named NR038975, which was confirmed to be markedly upregulated in the Gene Expression Profiling Interactive Analysis (GEPIA) dataset and our independent cohort of GC tissues. We firstly characterized the full-length sequence and subcellular location of NR038975 in GC cells. Our data demonstrated that upregulated NR038975 expression was significantly related to lymph node metastasis and TNM stage. In addition, knockdown of NR038975 inhibited GC cell proliferation, migration, invasion, and clonogenicity and *vice versa*. Mechanistically, RNA pull-down and mass spectrometry assays identified the NR038975-binding proteins and NF90/NF45 complex, and the binding was also confirmed by RNA immunoprecipitation and confocal experiments. We further demonstrated that genetic deficiency of NR038975 abrogated the interaction between NF45 and NF90. Moreover, NF90 increased the stability of NR038975. Thus, NR038975-NF90/NF45 will be an important combinational target of GC. Finally, we detected NR038975 in serum exosomes and serum of GC patients. Our results indicated that NR038975 was a biomarker for gastric tumorigenesis. The current study demonstrated that NR038975 is a novel lncRNA that is clinically and functionally engaged in GC progression and might be a novel diagnostic marker and potential therapeutic target.

## Introduction

Gastric cancer (GC) is one of the most common cancers and has the third highest death rate in the world (1). The incidence of GC displays widespread geographical differences, in which China exhibits a higher incidence rate of GC than other countries (2, 3). Advanced GC patients usually have a poor prognosis because of the lack of specific biomarkers for early diagnosis and effective treatments (4, 5). Recent studies have revealed potential targets for drug treatment; however, the specific mechanisms of GC still require further exploration and improved comprehension (6–8).

Long noncoding RNAs (lncRNAs) are a category of noncoding transcripts larger than 200 nucleotides in length. Multiple studies have demonstrated that lncRNAs participate in tumor development (9, 10). Previous evidence has shown that lncRNAs control diverse biological processes, such as migration, proliferation, cell cycle, and apoptosis. Moreover, lncRNAs are commonly differentially expressed in numerous human diseases, including cancers (11–13). The molecular mechanisms of lncRNAs on biological functions are diverse and confusing and include serving as microRNA (miRNA) sponges (14, 15), acting as scaffolds to regulate protein–DNA or protein–protein interactions (16, 17), and serving as decoys to bind to proteins (18, 19). These results demonstrate that lncRNAs significantly regulate tumor progression and could be used as potential therapeutic targets. LncRNAs are specifically expressed in certain tissues and cells (20) and could develop into therapeutic targets or diagnostic biomarkers for specific tissues. Furthermore, several lncRNAs act as cis-regulatory elements for neighboring genes because of their location in the nucleus (21); hence, targeting lncRNAs could regulate certain gene loci (12). Taken together, these results inspire the study of lncRNAs in the treatment of cancer.

RNA-binding proteins (RBPs) are crucial regulators of gene expression (22). NF90 and NF45 are encoded by the *ILF3* and *ILF2* genes, respectively, and combined into a complex *via* DZF (domain associated with zinc fingers) (23). NF90 and NF45 are ubiquitously expressed in human tissues and are increased in diverse cancers, including GC (24–28). Knockdown of NF90 or NF45 was shown to disturb the biological function of various cell lines (29). NF90 contains arginine-glycine-rich (RGG/RG) regions and two double-stranded RNA-binding motifs (dsRBMs) to bind certain nucleic acids (30, 31). NF90 can regulate the stability of numerous transcripts, such as Tau, interleukin (IL)-2, MyoD, and vascular endothelial growth factor (VEGF) (32–34). Therefore, NF90 and NF45 are developing as prospective therapeutic targets in several diseases, especially in cancer.

In this study, we identified a novel lncRNA, NR038975, and characterized its full-length sequence and subcellular location in GC cells. Furthermore, we explored the biological functions of NR038975 in GC cells and its underlying mechanisms. The results indicated that NR038975 was a novel lncRNA in GC progression. From the perspective of mechanism, NR038975 directly binds to NF45 and NF90 to promote the stability of the NF45/NF90 complex. Moreover, we detected the NR038975 expression in serum and serum exosomes. Overall, this study proposed insights on the biological roles and mechanisms of NR038975 in GC and further uncovered a novel diagnostic marker and therapeutic target in GC.

## Materials and Methods

### Clinical Specimens

Fresh GC tissues and adjacent normal gastric tissues in GC patients were collected at the Fourth Hospital of Hebei Medical University between 2015 and 2018 (n = 84). Informed consent was obtained from all patients for the use of the samples, and approval was obtained from the ethics committee of the Fourth Hospital of Hebei Medical University. All of the samples were diagnosed by 2–3 experienced pathologists. The inclusion criteria were primary GC at stages I–IV, and patients received surgery as the initial treatment.

### Cell Culture and Transfection

The GC cell lines AGS, MGC-803, and BGC-823 were obtained from the Shanghai Institute for Biological Sciences. The SGC-7901 cell line was acquired from GeneChem (Shanghai, China). The immortalized normal gastric epithelial cell line GES-1 was purchased from Procell Life Science & Technology (Wuhan, China). All of the cell lines were cultured in RPMI 1640 (Gibco, USA) supplemented with 10% fetal calf serum (BI, Israel) and penicillin and streptomycin (Invitrogen, USA) and incubated at 37°C with 5% CO_2_. Cells were transfected with the overexpression plasmid or vector (Generay, Shanghai, China). The vector was a negative control, and its structure was pCDH-CMV-MCS-EF1-CD511B-1 lentivector, as shown in **Supplementary Material**. The cells were transiently knocked down with small interfering RNA (siRNA; GenePharma, Shanghai, China).

### RNA Isolation and Quantitative Real-Time PCR

Total RNA of cells or tissues was extracted using TRIzol solution (Invitrogen, USA). Reverse transcription reactions were performed with reverse transcriptase (Promega, USA) according to the manufacturer’s instruction. Real-time PCR was conducted with SYBR Green PCR Kit (Promega, USA) in a Real-time PCR System (Bio-Rad, USA), and the gene-specific primers are shown in **Supplementary Table S1**. Relative expression levels of genes were calculated using the 2^-ΔΔCt^ method.

### 5′ and 3′ Rapid Amplification of cDNA Ends Analysis

To obtain the sequence of full-length lncRNA NR038975, 3′ rapid amplification of cDNA ends (RACE) and 5′ RACE analyses were performed using the GeneRacer™ Kit (Invitrogen) according to the manufacturer’s instructions. The specific primers are shown in the **Supplementary Table S1**.

### Cell Proliferation Assay

The proliferation of GC cells was measured in real time using the xCELLigence Real-Time Cell Analyzer (RTCA)-MP system (Acea Bioscience/Roche Applied Science). First, 100 μl of complete RPMI 1640 medium was added to each well of E-Plate 96 (Roche Applied Science) for equilibration. Then, 2 × 10^3^ cells in 100 μl of complete RPMI 1640 medium were added to each well. The E-Plate 96 was locked in the RTCA-MP device and continually cultured at 37°C with 5% CO_2_. The cell index directly reflects cellular proliferation.

### Migration and Invasion Assays

The cell migration assay was performed using 24-well Transwell filters (Corning Costar, USA). Here, 1 × 10^5^ SGC-7901 cells or MGC-803 cells in 0.2-ml serum-free medium were seeded in the upper chamber, while 0.6 ml of medium with 20% fetal bovine serum (FBS) was added to the lower chamber. After incubating for 15–16 h, the cells migrating through the filter were fixed with methanol, stained with crystal violet, and counted with a microscope in five randomly selected fields.

For the cell invasion assay, the transwell chambers were precoated with 200 μg/ml Matrigel (Beyotime Biotechnology, China) and incubated overnight. Here, 1 × 10^5^ SGC-7901 cells or MGC-803 cells in 0.2 ml serum-free medium were seeded in the upper chamber, while 0.6 ml of medium with 20% FBS was added to the lower chamber. After incubating for 20–22 h, the cells invading through the filter were fixed with methanol, stained with crystal violet, and counted with a microscope in five randomly selected fields. Data were obtained from three independent experiments.

### Clone Assay

To investigate the clonogenicity ability of the cells, 1 × 10^3^ cells with and without NR038975 knockdown and transfected with either NR038975 or vector were seeded into six-well plates and incubated for 10 days with medium changed every 3–4 days. Colonies were fixed with methanol for 10 min, stained with crystal violet, and observed and counted under a microscope.

### RNA Pull-Down Assay

Full-length sense and antisense NR038975 or its fragments were linearized with the corresponding restriction enzymes BamHI or EcoRI and transcribed *in vitro* with the Biotin-RNA Labeling Mix (Roche Diagnostics, USA). After obtaining whole lysates from MGC-803 cells, pull-down assays were performed, as described in a previous article (35). Then, the pulled down proteins were subjected to 10% sodium dodecyl sulfate-polyacrylamide gel electrophoresis (SDS-PAGE) and visualized by silver staining or immunoblotting assay. Protein bands were excised and identified by in-gel trypsin digestion followed by mass spectrometry (Capitalbio Corporation).

### RNA Immunoprecipitation

RNA immunoprecipitation (RIP) assays were performed using the Magna RIP™ RNA-Binding Protein Immunoprecipitation Kit (Merck Millipore) according to the manufacturer’s instructions. Then, the coprecipitated RNAs were quantified through quantitative RT-PCR. The primers used for detecting NR038975 are shown in **Supplementary Table S1**.

### Protein Immunoprecipitation

GC cells with stable knockdown of NR038975 were harvested and lysed in immunoprecipitation (IP) buffer (20 mM Tris, 1.5 mM MgCl_2_, 100 mM NaCl, 20 mM KCl, and 1% NP40, pH 7.6) with protease inhibitors (Roche). Lysates were placed on ice for 30 min, mixed every 10 min, and then cleared by centrifugation at maximum speed for 15 min at 4°C. Antibodies (Abs) for immunoprecipitation (2 µg NF90 and 2 µg normal rabbit IgG) were incubated with lysates for 8 h at 4°C with rotation. Protein G Dyna beads were added for 6 h at 4°C with rotation. Immunoprecipitates were washed six times with IP buffer and were used for Western blot.

### Western Blot Analysis

Transfected cells were lysed with lysis buffer containing protease inhibitors (Roche) at 4°C. Proteins were quantified using a bicinchoninic acid (BCA) protein assay kit (Thermo). Protein extracts were separated by 10% SDS-PAGE and transferred to polyvinylidene fluoride (PVDF) membranes. The membranes were blocked with 5% nonfat dry milk in TBS buffer for 1 h at room temperature and then incubated with antibodies at 4°C overnight. After washing with TBST, the membranes were incubated with horseradish peroxidase (HRP)-conjugated anti-mouse IgG or anti-rabbit IgG secondary antibody, and bands were detected using ChemiDoc™ XRS^+^ (Bio-Rad, USA) and ImageQuant™ LAS 4000 (GE Healthcare Life Sciences, USA). GAPDH was used as a loading control. The primary antibodies were NF45 (ab154791), NF90 (19887-1-AP), E-cad (sc-7870), N-cad (sc-7939), MMP9 (3852s), vimentin (sc-32322), and glyceraldehyde 3-phosphate dehydrogenase (GAPDH; 10494-1-AP).

### Animal Experiments

Four-week-old male BALB/C-nude mice were purchased from Vital River Laboratory Animal Technology, Beijing. To evaluate the effect of NR038975 *in vivo*, the animals were divided into the following two groups: SGC-7901 stably transfected with NR038975 shRNA and SGC-7901 control. All mice were subcutaneously injected in the left backside region with 1.5 × 10^6^ NR038975-knockdown SGC-7901 cells and the right backside region with 1.5 × 10^6^ control SGC-7901 cells. After 2 weeks, the tumor volumes were measured (V = 0.5 × length × width^2^) every 3 days. All the mice were sacrificed 4 weeks after inoculation, and tumors were excised, measured, weighed, and photographed.

### Statistical Analysis

Statistical analyses were performed using the SPSS 13.0 software. All data were obtained from three independent experiments, and each experiment was measured in triplicate. Quantitative data are presented as the mean ± standard deviation and were analyzed using the Student’s t-test or one-way ANOVA. Categorical data were expressed using proportions and compared with the chi-square test. A *p* value less than 0.05 was considered statistically significant (^*^p value <0.05), and all statistical tests were two-tailed.

## Results

### Identification of the Novel Gastric Cancer-Associated LncRNA NR038975

**Figure 1:**
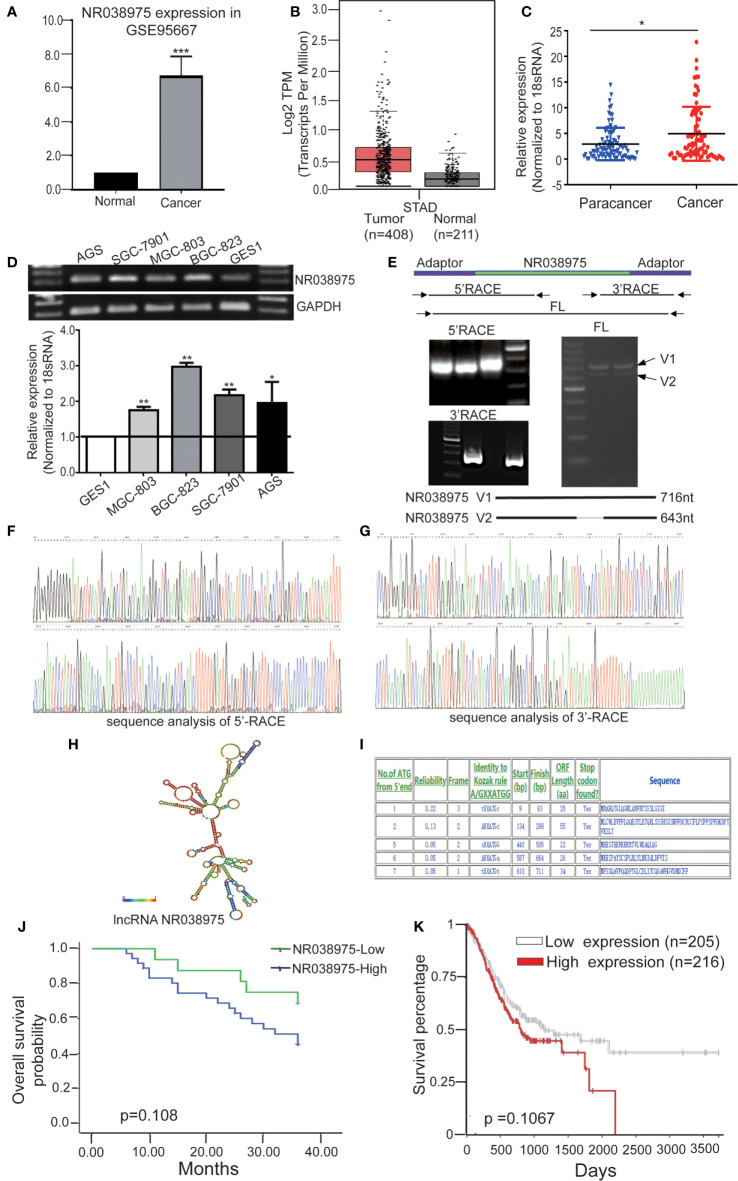
Identified the novel gastric cancer-associated long noncoding RNA (lncRNA) NR038975. **(A)** The expression of lncRNA NR038975 in the lncRNA/messenger RNA (mRNA) microarray analysis. **(B)** Relative expression of NR038975 in gastric cancer (GC) and normal tissues in Gene Expression Profiling Interactive Analysis (GEPIA). **(C)** NR038975 expression was analyzed by qRT-PCR in GC samples and para-cancer tissues (n = 84). Statistical analysis was performed using t-test. The horizontal line indicates the median value. *p < 0.05. **(D)** NR038975 expression was analyzed by RT and qRT-PCR in GC cells and gastric normal epithelial cells. **(E)** Rapid amplification of cDNA ends (RACE) analysis of NR038975 transcripts resulted in two RNA variants (V1:716 bp and V2:643 bp), and full-length (FL) V1 and V2 were obtained by RT-PCR. **(F)** Sequence analysis of 5′ RACE of NR038975. **(G)** Sequence analysis of 3′ RACE of NR038975. **(H)** NR038975 folds in a stable stem-loop structure. **(I)** NR038975 lacks the potential to encode any recognizable protein domains. **(J, K)** The effect of SNHG5 expression level on clinical prognosis was analyzed by Kaplan–Meier survival analysis using patient overall survival (OS) data in our sample cohort **(J)** and TCGA data **(K)**. **p < 0.01, ***p < 0.001.

Based on our previous lncRNA/messenger RNA (mRNA) microarray analysis of GC-associated lncRNAs (GSE95667) (36), which contains four pairs of GC tissues and normal tissues, lncRNA NR038975 was significantly upregulated in GC tissues compared with normal tissues (**Figure 1A**). In addition, we found that NR038975 was highly expressed in GC tissues compared with normal tissues in the Gene Expression Profiling Interactive Analysis (GEPIA) dataset (**Figure 1B**). We then detected the NR038975 expression in our independent cohort including 84 paired freshly frozen GC tissues and para-cancer tissues. NR038975 was remarkably overexpressed in GC tissues and showed a positive correlation with patient tumor size, TNM stage, and lymph node metastasis (**Figure 1C** and **Table 1**; p < 0.05). Furthermore, we quantified the NR038975 expression in the immortalized gastric epithelial cell line GES-1 and four GC cell lines. Significantly, high expression of NR038975 was found in the GC cell lines compared with GES-1 cells (**Figure 1D**). Our findings demonstrated that NR038975 might function as an oncogenic lncRNA in GC development.

**Table 1:** Correlation between NR038975 expression and clinicopathologic features in 80 GC patients.

Feature	NR038975		Chi-square	p value
	Low	High		
Age			0.514	0.473
<60	14	23		
≥60	13	30		
Gender			0.029	0.864
Male	22	44		
Female	5	9		
Tumor size			4.529	0.033^a^
<5	19	24		
≥5	8	29		
TNM stage			8.715	0.003^a^
I, II	13	9		
III, IV	14	44		
Differentiation			2.650	0.104
Well, moderate	12	14		
Poor	15	39		
T stage			9.638	0.002^a^
T1, T2	12	7		
T3, T4	15	46		
Lymph node metastasis			7.486	0.006^a^
Absent	13	10		
Present	14	43		

a These values have statistically significant differences.

GC, gastric cancer.

To determine the full-length transcript of NR038975, 5′ RACE and 3′ RACE were performed according to the manufacturer’s instructions (**Figures 1E–G**). As a result, we identified the following two NR038975 variants: one was 643 bp and the other was 716 bp, and they shared the same 5′ and 3′ ends. Interestingly, the expression of longer variant appeared to be more abundant in cells and thus became the focus of our further functional analysis (**Figure 1E**). In addition, we revealed that NR038975 formed a stable stem-loop structure by conducting theoretical RNA structure analysis (http://rna.tbi.univie.ac.at/) (**Figure 1H**). It was identified as an lncRNA rather than a protein-coding transcript based on a BLASTX analysis from the NCBI and ATGpr (http://atgpr.dbcls.jp/) websites (**Figure 1I**). Moreover, Kaplan–Meier survival analysis was conducted to determine the correlation of NR038975 expression with survival. The results demonstrated that higher NR038975 expression in patients was correlated with shorter survival, which could be confirmed in our sample cohort (**Figure 1J**) and The Cancer Genome Atlas (TCGA) dataset (**Figure 1K**). Taken together, these results firstly established that NR038975 was a novel lncRNA in GC cells and might be important in GC occurrence and development.

### LncRNA NR038975 Promotes the Proliferation, Migration, Invasion, and Clonogenicity of Gastric Cancer Cells

**Figure 2:**
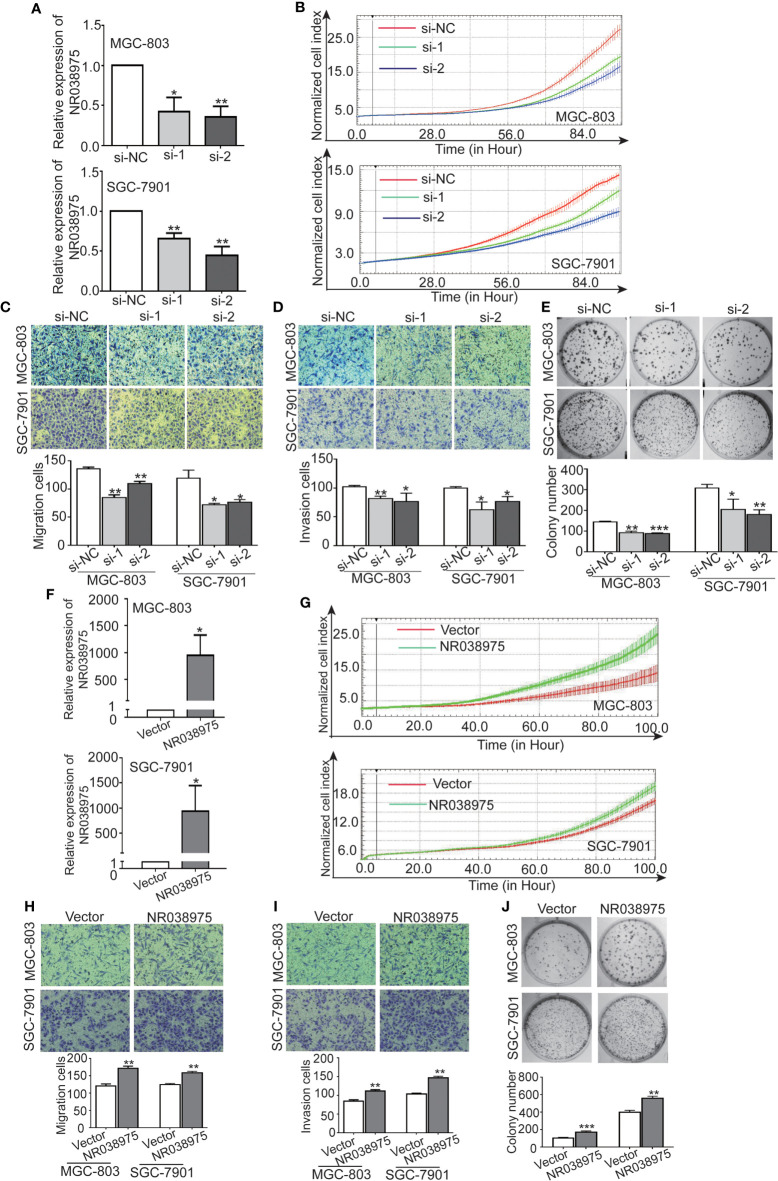
Inhibition of NR038975 reduced the proliferation, migration, invasion, and clonogenicity of gastric cancer (GC) cells. **(A)** The expression of NR038975 was detected by qRT-PCR in SGC-7901 cells and MGC-803 cells transfected with the small interfering RNA (siRNA) for 24 h, *p < 0.05. **(B)** Proliferation of GC cells with NR038975 knockdown was assessed by Real-Time Cell Analyzer (RTCA) system. **(C, D)** The effect of NR038975 knockdown on the migration and invasion of GC cells was investigated using the Transwell and Matrigel assay, respectively. Average counts were collected from five random microscopic fields, *p < 0.05. **(E)** Knocking down NR038975 inhibited the clonogenicity of GC cells; the number of colonies was calculated and plotted on a histogram, *p < 0.05. **(F)** The expression of NR038975 was detected by qRT-PCR in SGC-7901 and MGC-803 cells transfected with the plasmid for 24 h, *p < 0.05. **(G)** Proliferation of GC cells with NR038975 overexpression was assessed by RTCA system. **(H, I)** The effect of NR038975 overexpression on the migration and invasion of GC cells was investigated using the Transwell and Matrigel assay, respectively, *p < 0.05. **(J)** The effect of NR038975 overexpression on the clonogenicity of GC cells, *p < 0.05, **p < 0.01, ***p < 0.001.

We next investigated the biological function of NR038975 in GC cells. Because NR038975 was expressed at high levels and was easily transfected into SGC-7901 and MGC-803 cells, these cell lines were chosen for functional experiments (**Figure 1D**). First, we transiently transfected SGC-7901 and MGC-803 cells with NR038975 siRNAs or control. The NR038975 expression level was remarkably decreased in SGC-7901 and MGC-803 cells compared to that of the control group (**Figure 2A**). Furthermore, cell growth and transwell assays demonstrated that downregulation of NR038975 inhibited the proliferation (**Figure 2B**), migration (**Figure 2C**), and invasion (**Figure 2D**) of GC cells. In addition, NR038975-knockdown GC cells showed a decreased clonogenicity ability (**Figure 2E**). We further overexpressed NR038975 in SGC-7901 and MGC-803 cells with plasmids, and the expression level was significantly increased (**Figure 2F**). In contrast, overexpression of NR038975 promoted GC cell proliferation, migration, invasion, and clonogenicity (**Figures 2G–J**). Altogether, these results clearly demonstrated that NR038975 played a vital role in GC cell proliferation, migration, invasion, and clonogenicity.

### LncRNA NR038975 Promotes Gastric Cancer Growth *In Vivo*

**Figure 3:**
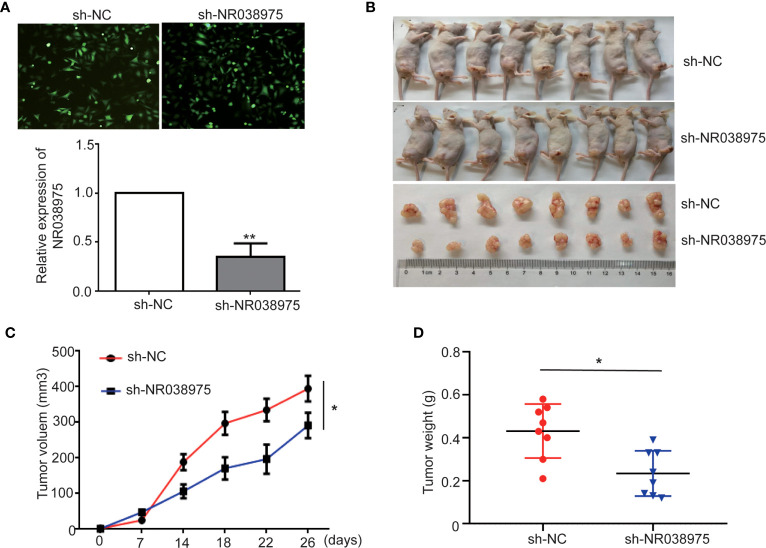
Knocking down NR038975 inhibits gastric cancer (GC) cell growth *in vivo*. **(A)** The stable and low expression of NR038975 was detected in SGC-7901 GC cells using immunofluorescence microscopy and real-time PCR after transfection with a lentivirus harboring the full-length human NR038975 sequence. **(B)** The growth of sh-NC-SGC-7901 cells and sh-NR038975-SGC-7901 cells that were injected subcutaneously into nude mice (n = 8). **(C)** Growth curves for the xenograft NR038975-knockdown tumors, *p < 0.05. **(D)** Tumor weights of the xenograft NR038975-knockdown tumors were measured, *p < 0.05, **p < 0.01.

To evaluate the effect of NR038975 on GC development *in vivo*, SGC-7901 cells with stable knockdown of NR038975 were subcutaneously injected into nude mice (n = 8) (**Figures 3A, B**). Tumor volumes were measured every 3 days. Mice were sacrificed after 26 days, and tumors were excised to calculate their volume and weight. As shown in **Figures 3C, D**, tumors in the SGC-7901 with NR038975-shRNA group were significantly smaller in size and weight than those in the control group. Taken together, lncRNA NR038975 could also promote GC cell growth *in vivo*.

### LncRNA NR038975 Interacts With the RNA-Binding Protein NF90/NF45

**Figure 4:**
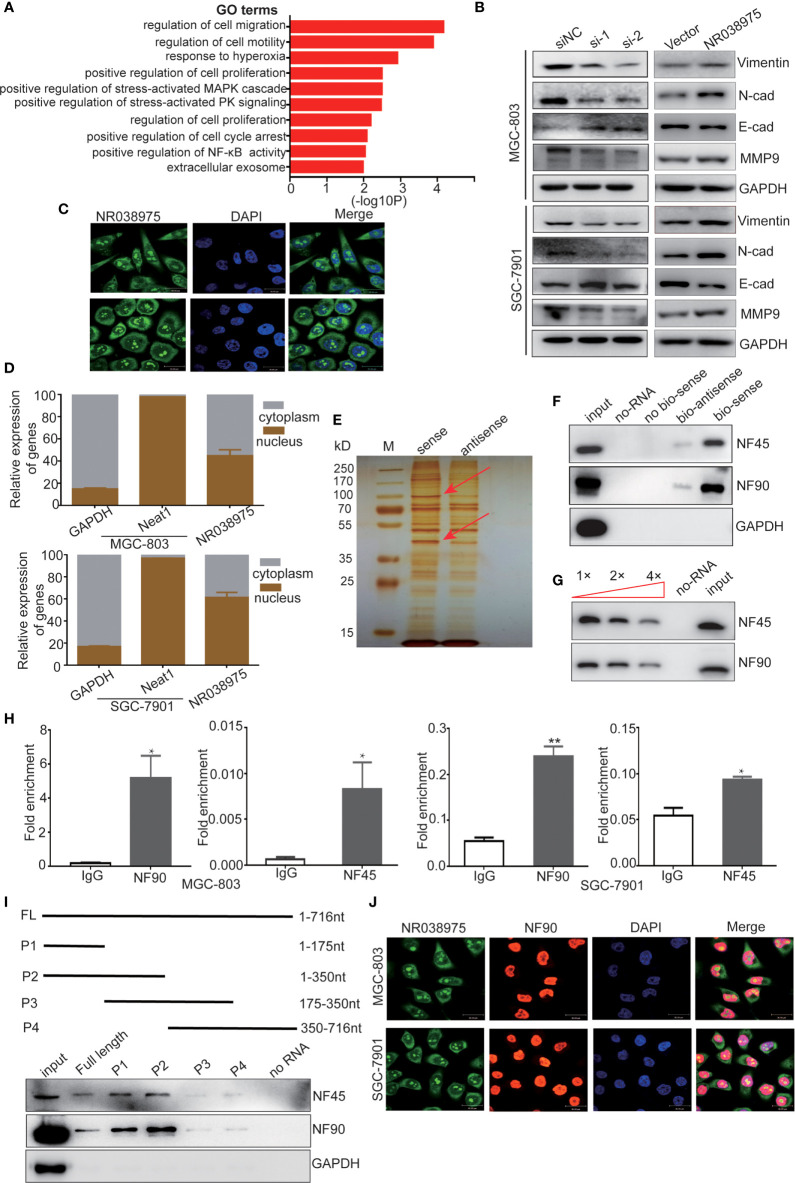
Long noncoding RNA (lncRNA) NR038975 binds to NF90 and NF45. **(A)** Gene Ontology analysis for all genes with altered expression after knockdown of NR038975. **(B)** Western blotting analysis of vimentin, N-cadherin, E-cadherin, and matrix metalloproteinase (MMP)9 in NR038975 knockdown and overexpressed gastric cancer (GC) cells. **(C)** Fluorescence *in situ* hybridization (FISH) of cells was performed using an NR038975 probe (green). The nuclei were counterstained using 4',6-diamidino-2-phenylindole (DAPI) (blue) (scale bar: 30 μm). **(D)** Expression levels of NR038975 in cytoplasm and nucleus of GC cells. **(E)** Silver staining of biotinylated NR038975-associated proteins. Specific bands were excised and analyzed through mass spectrometry. **(F)** Western blot analysis of the specific association of NF90 and NF45 with NR038975 from RNA pull-down assay. **(G)** Specific association of NR038975 with NF90 and NF45 proteins is performed by competition assay. Gradient concentrations of unlabeled NR038975 were added to compete with biotin-labeled NR038975 in interacting with NF90 and NF45. **(H)** RNA immunoprecipitation (RIP) assays were performed using NF90 or NF45 antibodies. Specific primers were used to detect NR038975, and RIP enrichment was determined relative to an input control. **(I)** Schematics of the full-length and deletion fragments of NR038975 used for the precipitation of NF90 and NF45 from MGC-803 cell lysates. Associated NF90 and NF45 proteins were detected by Western blotting. **(J)** MGC-803 and SGC-7901 cells were seeded on glass coverslips in 24-well plates and then fixed with 4% paraformaldehyde. NR038975 probes (green) and a fluorescence-conjugated antibody against NF90 (red) were used for combined application of RNA-FISH and immunofluorescence. Cell nucleus was stained with DAPI (blue). *p < 0.05, **p < 0.01.

To explore the underlying regulatory mechanism of NR038975, we transfected MGC-803 cells with NR038975 siRNA or control and then performed RNA transcriptome sequencing. The data were submitted to the Sequence Read Archive (SRA), and the accession number is PRJNA669071. In which, 3,149 genes were significantly changed (|fold change| >1.5). Gene Ontology (GO) analysis revealed that the differentially expressed genes were enriched in cancer proliferation- and migration-related terms (**Figure 4A**). Then, we examined the expression of migration- and invasion-related proteins matrix metalloproteinase (MMP)9, vimentin, N-cadherin, and E-cadherin in NR038975 knockdown and overexpressed GC cells (**Figure 4B**).

To further investigate the mechanism of NR038975, we studied its cellular localization. We designed NR038975 probes and used RNA-fluorescence *in situ* hybridization (FISH) to demonstrate that NR038975 was located in both the cytoplasm and nucleus (**Figure 4C**). We also isolated the cytoplasmic and nuclear fractions of MGC-803 and SGC-7901 and conducted real-time PCR (**Figure 4D**). We found that NR038975 was located in the cytoplasm and nucleus almost equally, further confirming the above result.

An increasing number of studies have revealed that lncRNAs may function by physically interacting with specific proteins; therefore, RNA pull-down assay was performed to identify NR038975-interacting proteins. Biotinylated sense or antisense NR038975 was incubated with MGC-803 cell lysate, pulled down with streptavidin, and analyzed by SDS-PAGE and silver staining (**Figure 4E**). Among the identified proteins, NF45/ILF2 was the most enriched NR038975-binding partner. Meanwhile, NF90/ILF3, which was dimerized with NF45 to form a complex, was also identified. Furthermore, NF45 and NF90 were both detected in NR038975-specific pull-down cell lysates by Western blotting (**Figure 4F**).

To confirm the specific interaction between NF90/NF45 and NR038975, competition assay was performed by adding a gradient concentration of non-biotinylated NR038975. The interaction between biotinylated NR038975 and NF90/NF45 was competed by non-biotinylated NR038975 in a dose-dependent manner (**Figure 4G**). Moreover, we validated the interaction between NR038975 and NF90/NF45 by RIP. We detected a significant enrichment of NR038975 using NF90 and NF45 antibodies (**Figure 4H**). In addition, we performed deletion-mapping analysis to identify which region of NR038975 is required for NF90/NF45 binding. We found that transcripts containing 1-350nt exhibited the strongest binding to NF90/NF45 (**Figure 4I**). Furthermore, confocal microscopy was used to investigate the colocalization of NR038975 and NF90. We found that NR038975 and NF90 were located in the nucleus together (**Figure 4J**). Altogether, the RNA pull-down, RIP, deletion mapping, and colocalization assays demonstrated a direct interaction between NR038975 and NF90/NF45.

### LncRNA NR038975 Is a Molecular Linker Between NF45 and NF90, and NF90 Enhances NR038975 Stability in Gastric Cancer Cells

**Figure 5:**
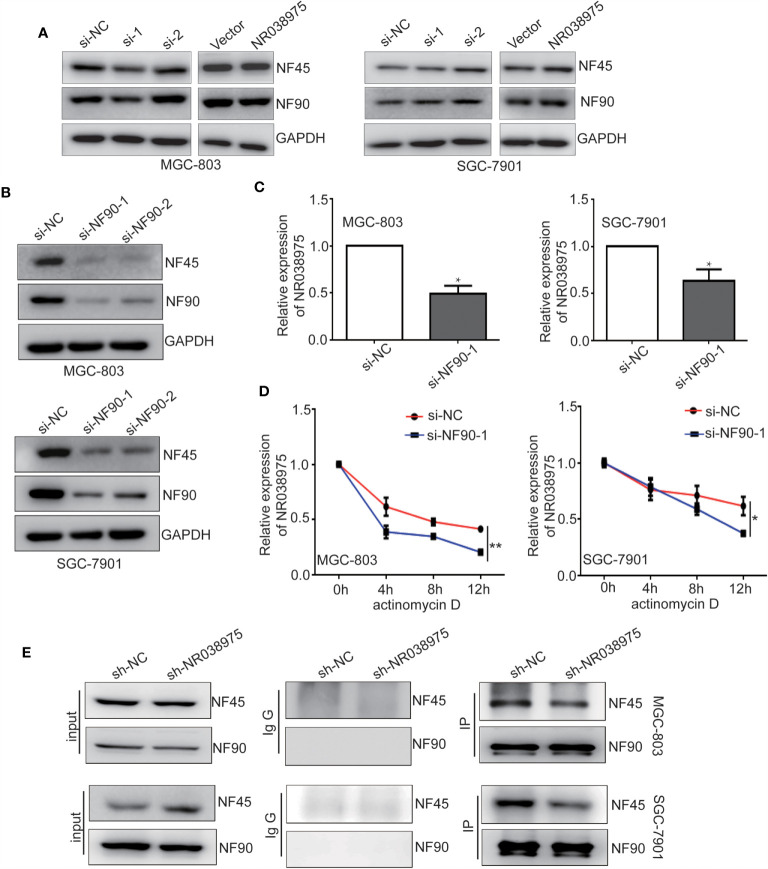
Long noncoding RNA (lncRNA) NR038975 directly links NF45 and NF90. **(A)** NF90 and NF45 expression levels were measured through Western blotting after knockdown or overexpression of NR038975. **(B)** NF90 and NF45 expression levels were measured through Western blotting after knockdown of NF90. **(C)** NR038975 expression level was measured by real-time PCR after knockdown of NF90, *p < 0.05. **(D)** The half-life of NR038975 after treatment with 5 μg/ml actinomycin D for indicated times, with NF90 knockdown in MGC-803 and SGC-7901 cells, *p < 0.05. **(E)** Immunoprecipitation assay was performed to detect the interaction between NF90 and NF45 in NR038975-stably knockdown MGC-803 and SGC-7901 cells, **p < 0.01.

Subsequently, we attempted to explore the functional relevance of the interaction between NR038975 and NF90/NF45. The results of the present study demonstrated that downregulation of NR038975 had no effect on the expression of NF90 and NF45 (**Figure 5A**). However, downregulation of NF90 significantly decreased the expression of NF45 and the RNA levels of NR038975 in MGC-803 and SGC-7901 cells (**Figures 5B, C**). To confirm the above findings, we treated GC cells with the RNA synthesis inhibitor actinomycin D and harvested RNAs at 0, 4, 8, and 12 h. We found that NR038975 degraded more quickly in the NF90 knockdown group than in the control group (**Figure 5D**), illustrating that NF90 specifically regulated the stability of NR038975 in GC cells.

In addition, NR038975 specifically binds NF90 and NF45 in GC cells, and NF90 and NF45 interact with each other. However, downregulation of NR038975 had no effect on the expression of NF90 and NF45. We speculated that NR038975 is important for NF90 and NF45 complex interactions. To verify this hypothesis, we performed an IP assay using NF90 antibody in MGC-803 and SGC-7901 cells stably transfected with NR038975 shRNA or control and found that NR038975 knockdown led to a significant decrease in the interaction of NF90/NF45 (**Figure 5E**). Taken together, we are the first to find that NR038975 directly linked NF45 and NF90, which regulated the function of the NF45/NF90 complex.

### LncRNA NR038975 Functions Through Its Interaction With NF90

**Figure 6:**
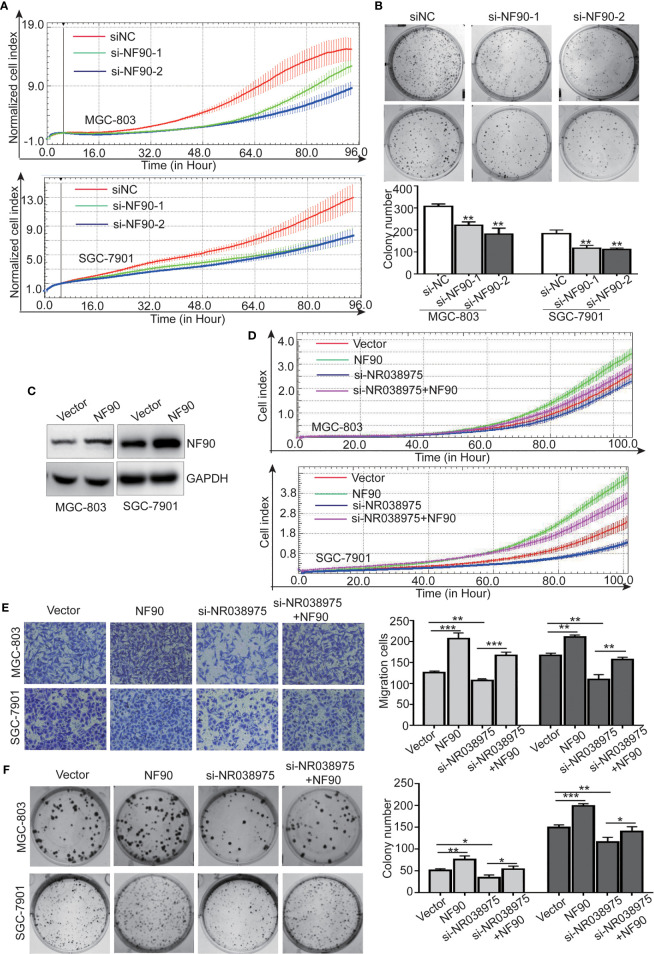
Long noncoding RNA (lncRNA) NR038975 functions through its interaction with NF90. **(A)** Proliferation of MGC-803 and SGC-7901 cells with knocked down NF90 was assessed by Real-Time Cell Analyzer (RTCA) system. **(B)** The effect of NF90 knockdown on colony formation in gastric cancer cells was investigated. **(C)** NF90 expression level was measured through Western blotting after overexpression of NF90. **(D–F)** Overexpression of NF90 reversed the NR038975 knockdown-reduced growth **(D)**, migration **(E)**, and colony formation **(F)** of MGC-803 and SGC-7901 cells. *p < 0.05, **p < 0.01, and ***p < 0.001.

To determine the function of NF90 in GC, we knocked down NF90 expression in SGC-7901 and MGC-803 cells. The results revealed that downregulation of NF90 remarkably inhibited the growth and clonogenicity of GC cells (**Figures 6A, B**), which demonstrated that NF90 could promote the development of GC.

Furthermore, to explore whether the NR038975-mediated biological function in GC cells depended on its binding with NF90, we constructed MGC-803 and SGC-7901 cells with an NF90 overexpression plasmid (**Figure 6C**). The results demonstrated that downregulation of NR038975 inhibited the proliferation, migration, and clonogenicity of GC cells, which were rescued by overexpression of NF90 (**Figures 6D–F**). Collectively, these results illustrated that the mechanism by which NR038975 operates in GC cells is partly attributed to the NR038975/NF90/NF45 association complex.

### NR038975 Expression Is Elevated in the Plasma and Plasma Exosomes of Gastric Cancer Patients

**Figure 7:**
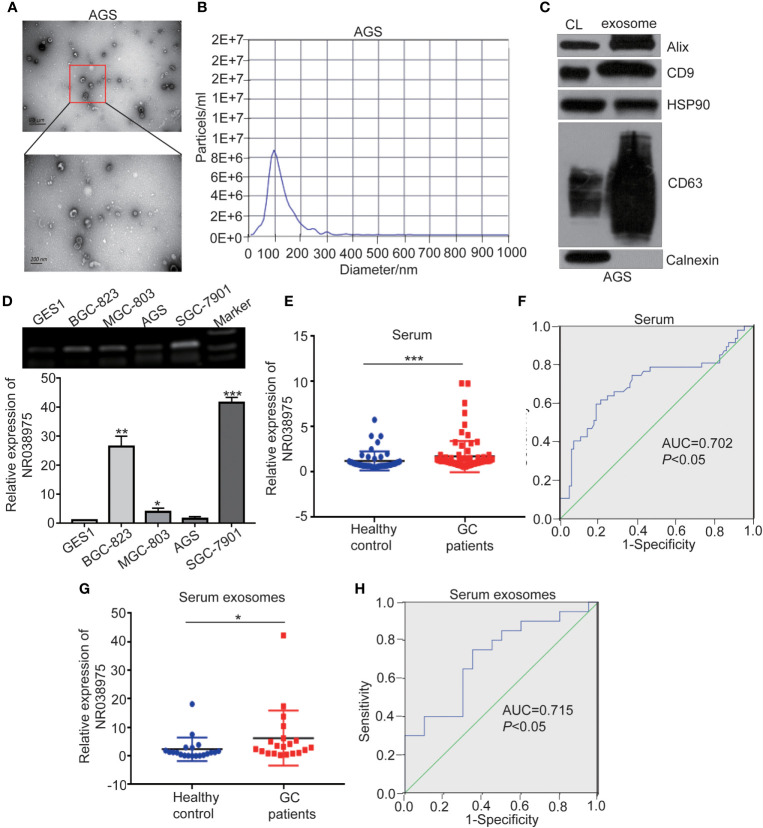
NR038975 exists in gastric cancer (GC) cell-derived exosomes and is upregulated in the serum of GC patients. **(A)** Representative TEM images of exosomes secreted by AGS cells. **(B)** Nanoparticle tracking analysis (NTA) of the size distributions and numbers of exosomes derived from AGS cells. **(C)** The markers of exosomes were detected by Western blot. **(D)** NR038975 expression in exosomes isolated from four GC cell lines and one immortalized normal gastric epithelial cell line (GES1) was detected by RT-PCR and qPCR. **(E)** NR038975 expression levels in serum of GC patients and healthy controls. **(F)** Receiver operating characteristic (ROC) curves for the diagnostic value of serum NR038975 in GC. **(G)** NR038975 expression levels in serum exosomes of GC patients and healthy controls. **(H)** ROC curves for the diagnostic value of serum exosomal NR038975 in GC. *p < 0.05, **p < 0.01, ***p < 0.001.

Recent studies have shown that lncRNAs could also be secreted into the blood and serve as diagnostic markers in cancer development. We attempted to determine whether NR038975 can be secreted by GC cells through exosomes and serve as a noninvasive biomarker for GC. Thus, we extracted exosomes from the culture supernatant of GC cell lines. The morphology and size distribution of exosomes were verified by transmission electron microscopy (TEM) and nanoparticle tracking analysis (NTA). We found that the exosomes were typical lipid bilayer membrane-encapsulated nanoparticles (**Figure 7A**) and had a predominant size of 50–150 nm (**Figure 7B**). In addition, the exosome marker proteins Alix, CD9, heat shock protein (HSP)90, and cluster of differentiation (CD)63 were detected in both cell lysate and exosomes; however, calnexin was only detected in the cell lysate (**Figure 7C**). Then, we measured NR038975 in GC cell-derived exosomes using RT-PCR and qPCR. The results showed that NR038975 was highly expressed in GC cell-derived exosomes but weakly expressed in exosomes of GES-1 cells (**Figure 7D**), illustrating that NR038975 could be secreted by GC cells.

Furthermore, we tested NR038975 expression in plasma samples (47 healthy controls and 86 GC patients) and used receiver operating characteristic (ROC) curve to analyze the diagnostic value of NR038975 as a biomarker for GC. We found that NR038975 expression was upregulated in the plasma of GC patients compared to healthy controls (**Figure 7E**), and the area under the ROC curve (AUC) was 0.702 (**Figure 7F**). We also isolated exosomes from the samples (20 healthy controls and 20 GC patients) and detected the expression levels of NR038975, which was increased in GC patients compared to healthy controls (**Figure 7G**). The AUC was 0.715 (**Figure 7H**). Altogether, the results suggested that NR038975 can be secreted by the exosomes of GC cells and might be a diagnostic marker for GC.

## Discussion

GC is one of the most common causes of cancer-related death worldwide. GC pathogenesis includes many mutations in tumor suppressor genes and amplifications of oncogenes (37–39). An increasing number of studies have revealed that lncRNA expression profile is considerably dysregulated in GC, and certain lncRNAs, such as TINCR, FENDRR, and GAPLINC, are related to tumorigenesis (40–42). Although dysregulation of specific lncRNAs in gastric tumorigenesis is a recognized phenomenon, the functional mechanisms of most lncRNAs remain debatable in human GC. A growing number of underlying mechanisms of lncRNAs involved in GC could pave the way for novel therapies to overcome the disease.

**Figure 8:**
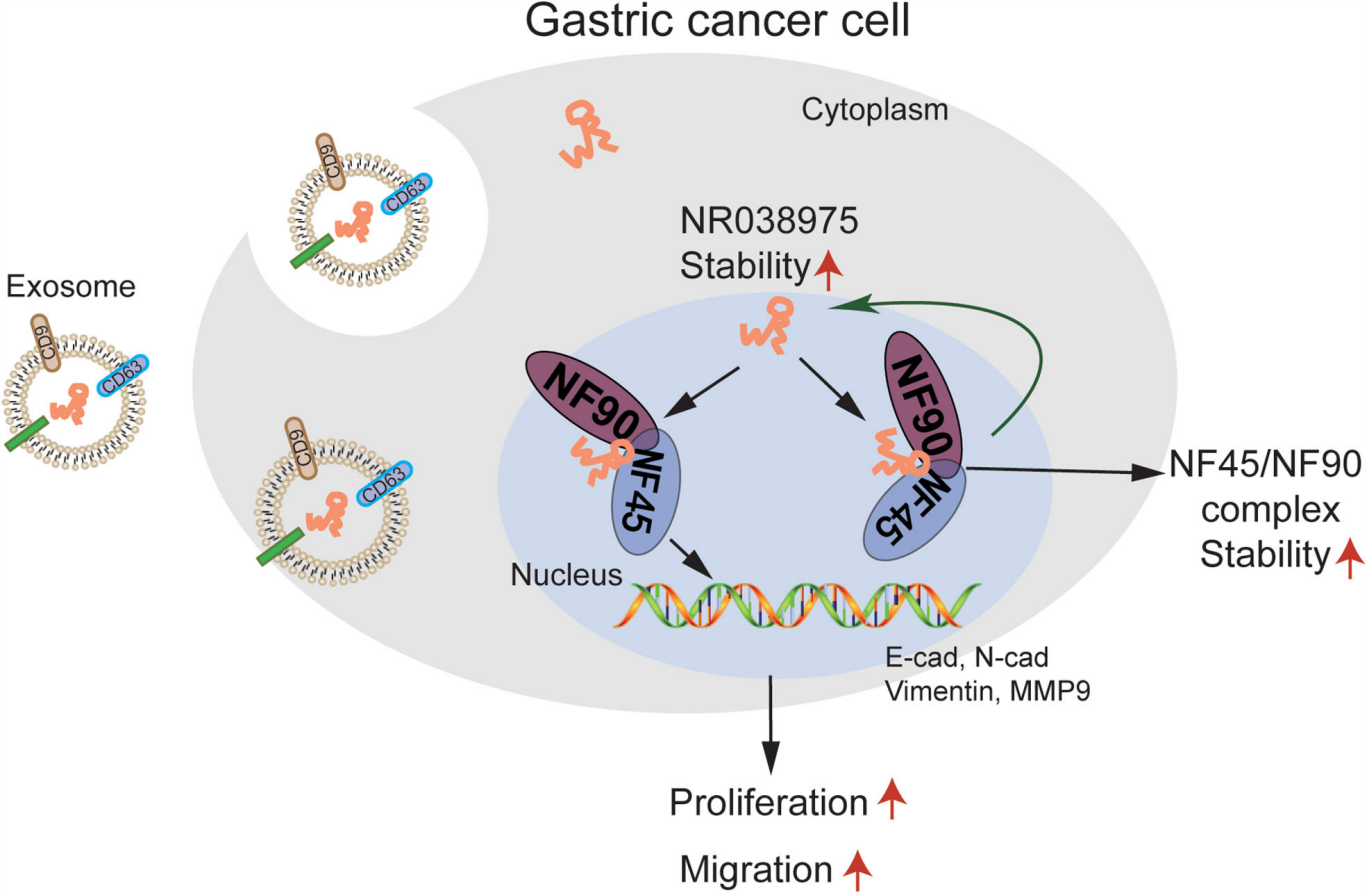
A schematic model of long noncoding RNA (lncRNA) NR038975 functions in gastric carcinogenesis. NR038975 directly binds to NF45 and NF90, which not only increases the stability of NR038975 but also increases the stability of NF45/NF90 complex, so as to promote gastric cancer proliferation and migration. Moreover, NR038975 could be secreted to serum exosome as a diagnostic marker.

In the present study, we first found that NR038975 was upregulated in GC tissues. The biological functions and specific mechanism of NR038975 in GC development have never been illuminated. We revealed that the NR038975 expression level was significantly related to TNM stage. These results also showed that NR038975 dramatically promoted GC cell proliferation, migration, invasion, and clonogenicity. NR038975 directly binds to NF45 and NF90, which not only increases the stability of NR038975 but also increases the stability of NF45/NF90 complex, so as to promote GC proliferation and migration. Moreover, NR038975 could be secreted to serum exosome as a diagnostic marker (**Figure 8**).

A growing number of lncRNAs have been reported to promote the progression of GC. For example, lncRNA GClnc1 acts as a modular scaffold of the WDR5 and KAT2A complexes to specify the histone modification pattern to promote gastric carcinogenesis (43). H19 and uc0011sz serve as novel biomarkers for the diagnosis of GC (44, 45). In our previous study, we identified the lncRNA GALNT5 uaRNA, which is highly expressed in GC. We found that GALNT5 uaRNA promoted GC progression through its interaction with HSP90 and decreased ubiquitination of protein kinase B (AKT) and inhibitor of nuclear factor kappa-B kinase (IKK) (36). Here, our study demonstrated that NR038975 could promote GC development. Knockdown of NR038975 in GC cells decreased tumor cell proliferation, migration, invasion, and clonogenicity *in vitro* and diminished tumor cell proliferation *in vivo*. In addition, transcriptome analysis also revealed that the main cellular functions targeted by NR038975 knockdown were related to oncogenesis, providing molecular insights into the role of NR038975 in tumor development. Further studies are required to verify how NR038975 regulates tumor progression at the transcriptional level.

Mechanistic studies about lncRNA–protein interactions have illustrated that lncRNAs could act as molecular scaffolds to link or gather several proteins and concertedly regulate gene expression (46). In this article, we performed an RNA pull-down assay followed by mass spectrometry analysis and found that NF45 and NF90 were the most enriched NR038975-binding proteins. Our results identified NR038975 as a novel binding partner for NF90 and NF45 that enhanced the interaction between NF90 and NF45. Studies have revealed that the NF90/NF45 complex serves as a multifunctional stimulator that integrates different steps of gene expression to accelerate the rapid response of inducible genes. The NF90/NF45 complex plays important roles in viral replication and RNA metabolism to regulate gene expression and mRNA stability (47, 48). In addition, NF90 and NF45 occupy the *c-fos* gene enhancer/promoter region and function as transcriptional coactivators (49). Nourreddine et al. (50) implied that the NF90/NF45 complex plays crucial roles in mitosis by competing with the staufen-mediated mRNA decay (SMD) machinery for a common set of mRNAs. Taken together, the NF90/NF45 complex is a multifunctional factor in the development of diseases, and lncRNA NR038975 promotes GC tumorigenesis by binding to NF90/NF45 and increasing the stability of the NF90/NF45 complex.

On the other hand, NF90 is a specific RNA-binding protein that leads to mRNA translation, stabilization, or degradation (33, 47). NF90 was reported to increase VEGF mRNA stability by combining with human antigen R (HuR) and heterogeneous nuclear ribonucleoprotein L (hnRNP L) (33) or regulate IL-2 mRNA stability by competing with destabilizing proteins (51). Furthermore, NF90 could be induced to degrade lncRNA-LET and then affect hypoxia-inducible factor (HIF)-1α expression (52). NF90 can also form a specific complex with VEGFA mRNA to promote its translation (53). In our study, we showed that knockdown of NF90 decreased the expression of NR038975. Thus, NR038975 was stabilized by binding to NF90. Therefore, the results suggested that upregulated levels of NF90 expression share a strong correlation with GC progression and development, while postulations have been made highlighting its ability as a potential therapeutic target in cancer.

LncRNAs not only are involved in tumorigenesis but also act as novel noninvasive biomarkers in patients, especially in body fluids (54). Exosomes contain various contents, including lncRNAs, which can be absorbed by adjacent cells and influence the function of recipient cells (55–57). LncRNA PCAT1 was reported to be highly expressed in esophageal squamous cell carcinoma (ESCC) cell-derived exosomes and ESCC patient serum and to enhance cell proliferation by sponging miR-326 (58). LncRNA UFC1 was upregulated in non-small cell lung cancer (NSCLC) tissues, serum exosomes, and serum, which promoted NSCLC by decreasing phosphatase and tensin homolog deleted on chromosome ten (PTEN) expression (59). Moreover, lncUEGC1 is identified to be significantly upregulated in plasma exosomes of early-stage GC patients and has better diagnostic accuracy (60). In this study, we showed that NR038975 was highly expressed in exosomes from GC cells and the serum of GC patients, indicating its potential role as a GC biomarker.

In conclusion, we demonstrated that NR038975 is significantly upregulated in tumor tissues and serum and serum exosomes of GC patients. Mechanistically, we identified the NF90/NF45 complex as a functional NR038975-binding partner, providing an alternative strategy for drug development by targeting the NR038975–NF45/NF90 interaction. These novel findings make NR038975 a promising diagnostic marker and therapeutic target in GC.

## Data Availability Statement

The datasets presented in this study can be found in online repositories. The names of the repository/repositories and accession number(s) can be found in the article/**Supplementary Material**.

## Ethics Statement

The studies involving human participants were reviewed and approved by The Ethics Committee of Fourth Hospital of Hebei Medical University. The patients/participants provided their written informed consent to participate in this study. The animal study was reviewed and approved by The Ethics Committee of Fourth Hospital of Hebei Medical University.

## Author Contributions

SW, SD, and LZ conceived the study. SW, SD, CZ, RZ, and ZZ carried out the experiments. YS, LZ, and BS analyzed the data. SW and SD wrote the article. All authors contributed to the article and approved the submitted version.

## Funding

This work was supported by the Natural Science Foundation of China (Nos. 81772550, 81673642, 81502032, 81973520, and 81902798), the Outstanding Youth Foundation of Hebei Province, China (H2019206697), and the Natural Science Foundation of Hebei Province (H2020206131).

## Conflict of Interest

The authors declare that the research was conducted in the absence of any commercial or financial relationships that could be construed as a potential conflict of interest.

## Publisher’s Note

All claims expressed in this article are solely those of the authors and do not necessarily represent those of their affiliated organizations, or those of the publisher, the editors and the reviewers. Any product that may be evaluated in this article, or claim that may be made by its manufacturer, is not guaranteed or endorsed by the publisher.
