# Supplementary material for: LncRNA NR038975, A Serum-Based Biomarker, Promotes Gastric Tumorigenesis by Interacting With NF90/NF45 Complex
Source: Front Oncol. 2021 Nov 17;11:721604. doi: 10.3389/fonc.2021.721604 (PMC8660099; doi:10.3389/fonc.2021.721604)
Supplement: Supplementary file 1 [file DataSheet_1.docx]

1. pCDH-CMV-MCS-EF1-CD511B-1 lentivector:


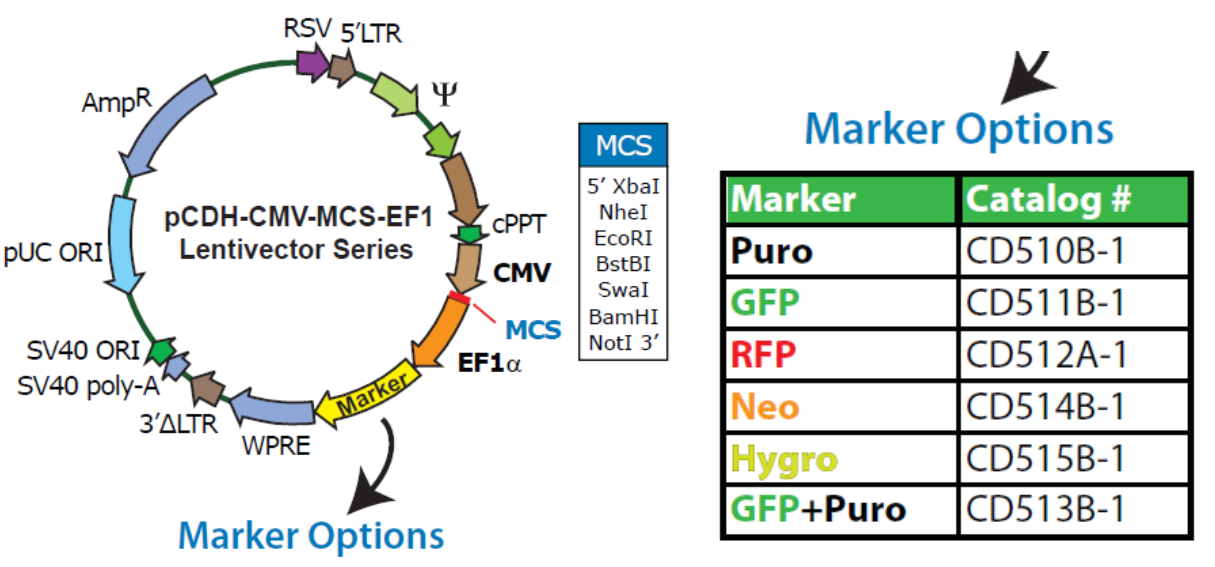


1. The cDNA sequence of NR038975:

ATAGTTCAATGCGAGCTGAGCAGACAGGGCTGCAAGGAAATCTGGCGCGGTTCAATACCTCGTCTAGCCTGGGTTCCAGTATCTAATTTTTTTTTTGTTTTAACTGACAAACTCATTTCTCTACTGGGACAGGATGCTGTGCTGGCTGGAAGTTCCATTTCTACAGCAAGAATCCTATCTGGAAACACAGAAGTTGTCCTCTAGCCACAGCAGCTCGAACTTTTTTGATTGTCGTTGCTGCTTTCTCCCATCACCCCCATCCCCTTTTGACAAAGATCCAACTGTAAAAAGTCTTACGTAACAGTTCAGGACTACTTCGGTTCTTTTACTGGATCATATAAATCTTTCGCCTTTTAAATCATGTATTAAAAAAAAAGAAAAAGGAAAAACAAAGATTTCTTTTAGATGCAGAATCTACCTACATTTGGCTGAAAATCAGATGGAAGAATCCACAGAGGAAAGGAAGGAAAGGCGAACTGTCCTGTGGAAAGCACAGCTGCAGGGATAGTAGAAAGTAACAGGCTCTCGATCCGTGGGAGGTGGTCACTTTGGAGGACAGATAAGTCATCCTCAGAGAACGAACACAATGAAGGAAATTCCAGCCTATTCATGTTCTCCTCTCAGGCTGTACCTCAACAAGATCCAACTGGACTTTGTGATCTCATAACCTGTCAAGGGGCCTGGCATGGAGTAGATAATAAATGTTTTTTTTAATC

1. The full uncropped and unedited version of western blots.

Figure 4

**bio-antisense**

g

f

**bio-sense**

**beas**

**input**

input

no RNA

1× 2× 4×


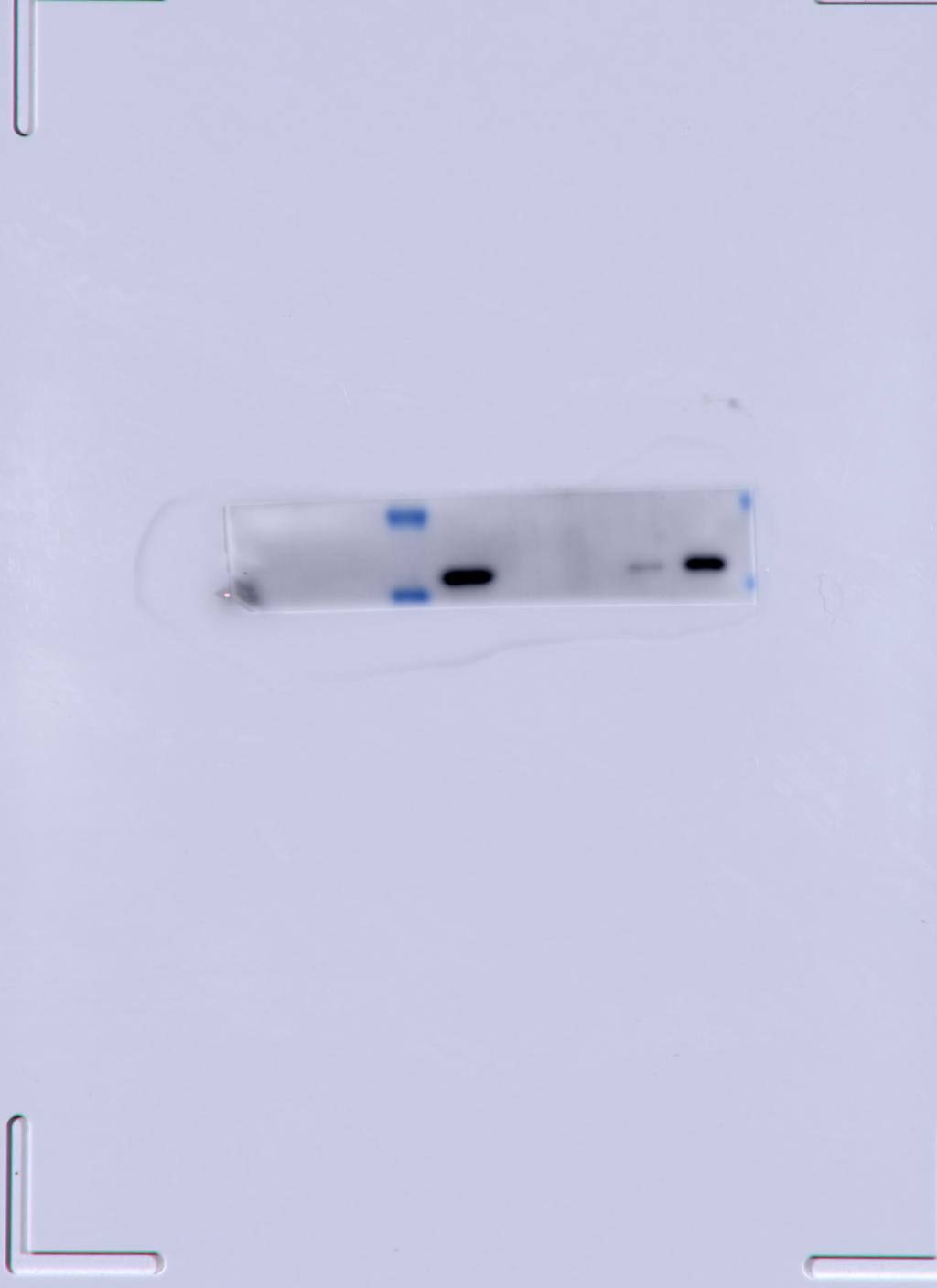


NF45


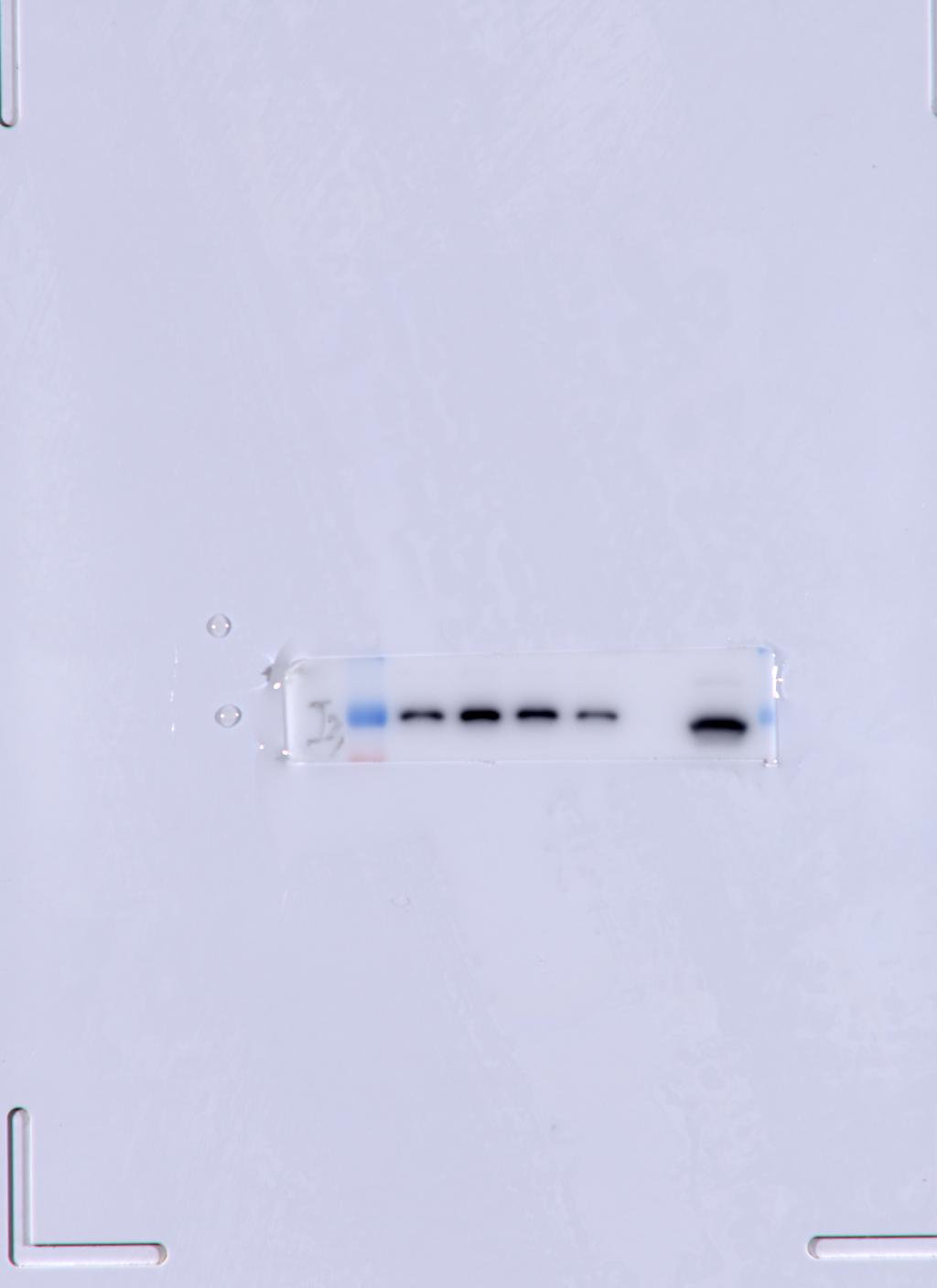


NF90

90kD

40kD


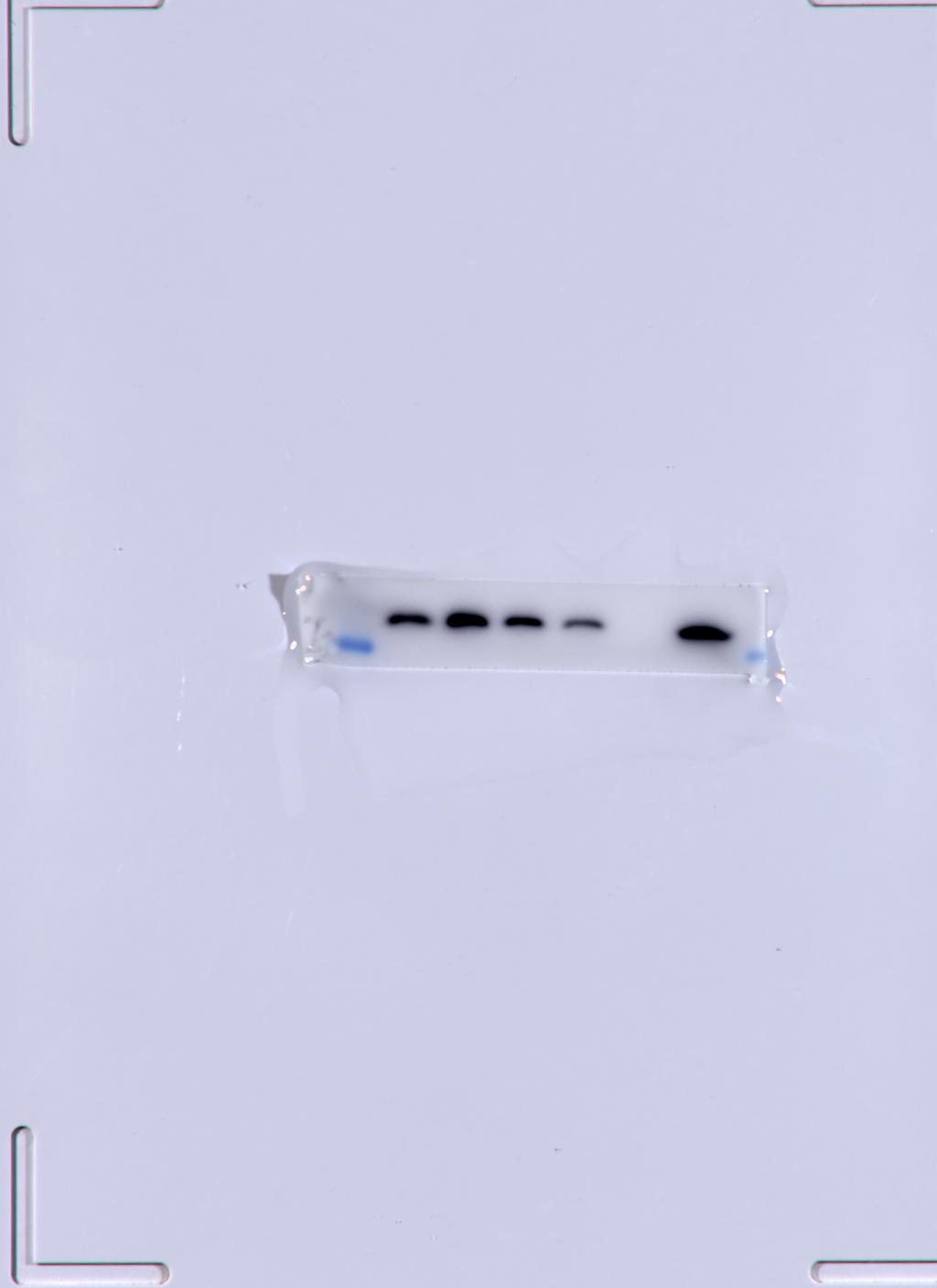

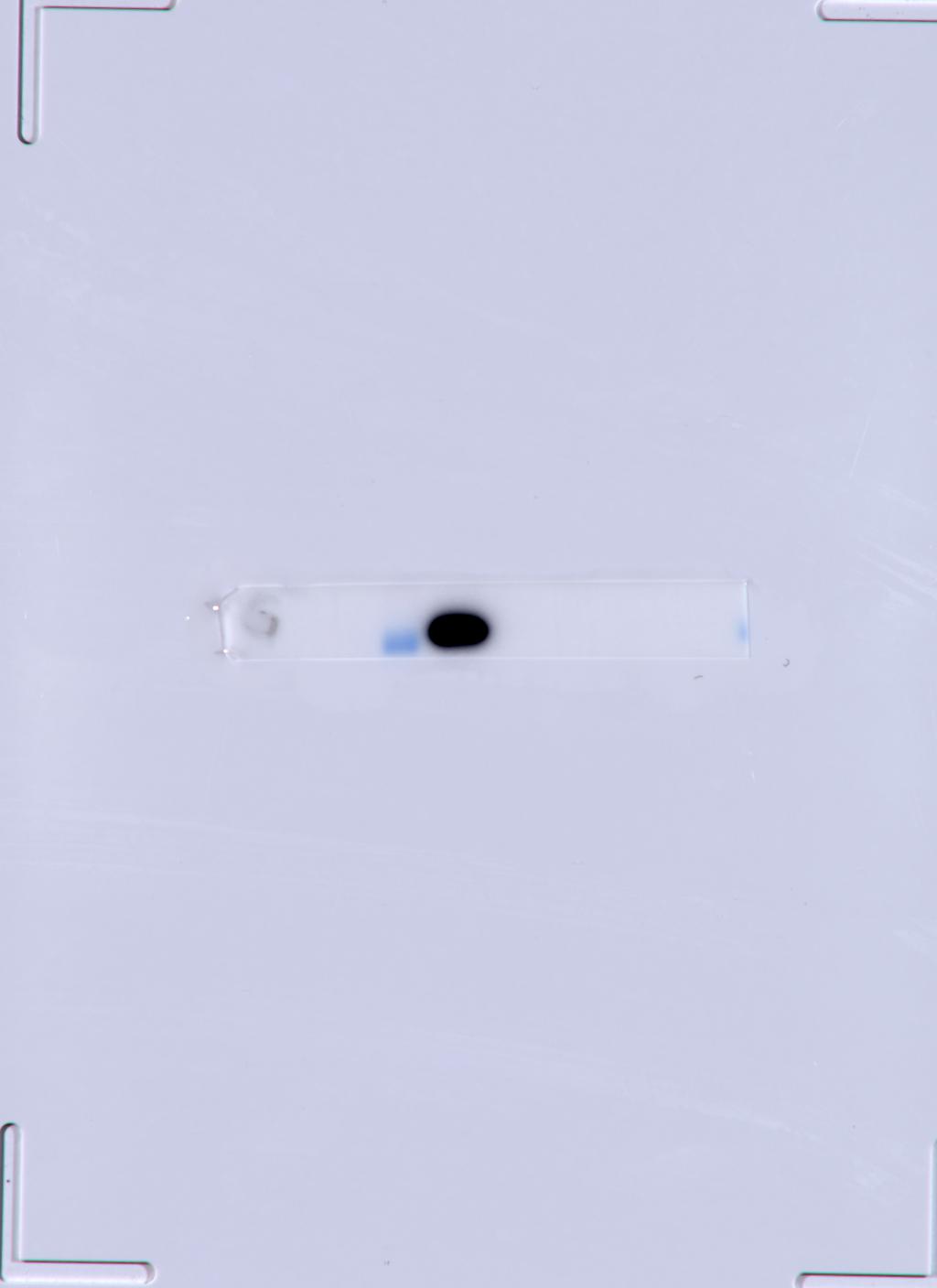

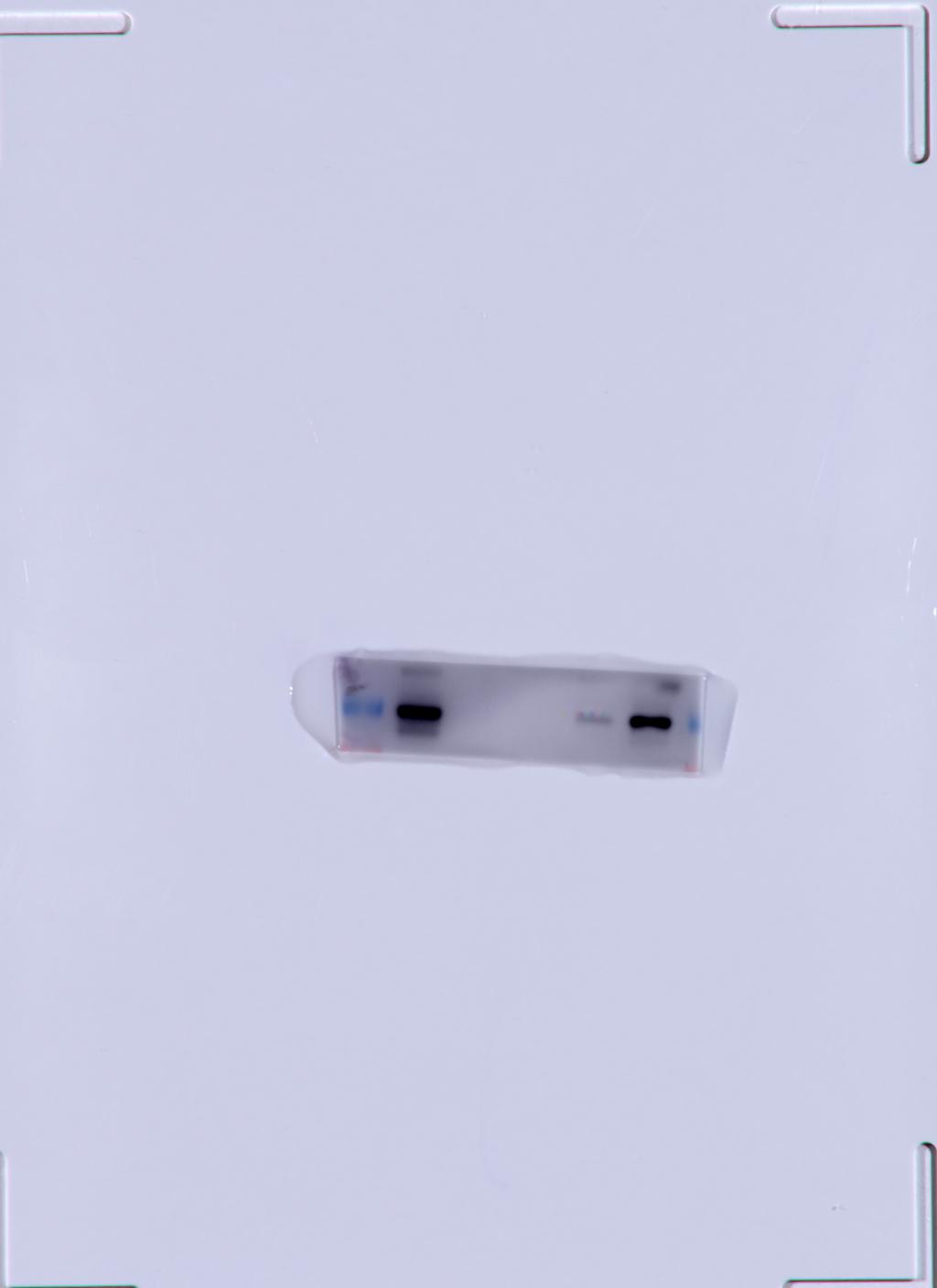


NF45

40kD

GAPDH

NF90

35kD

90kD




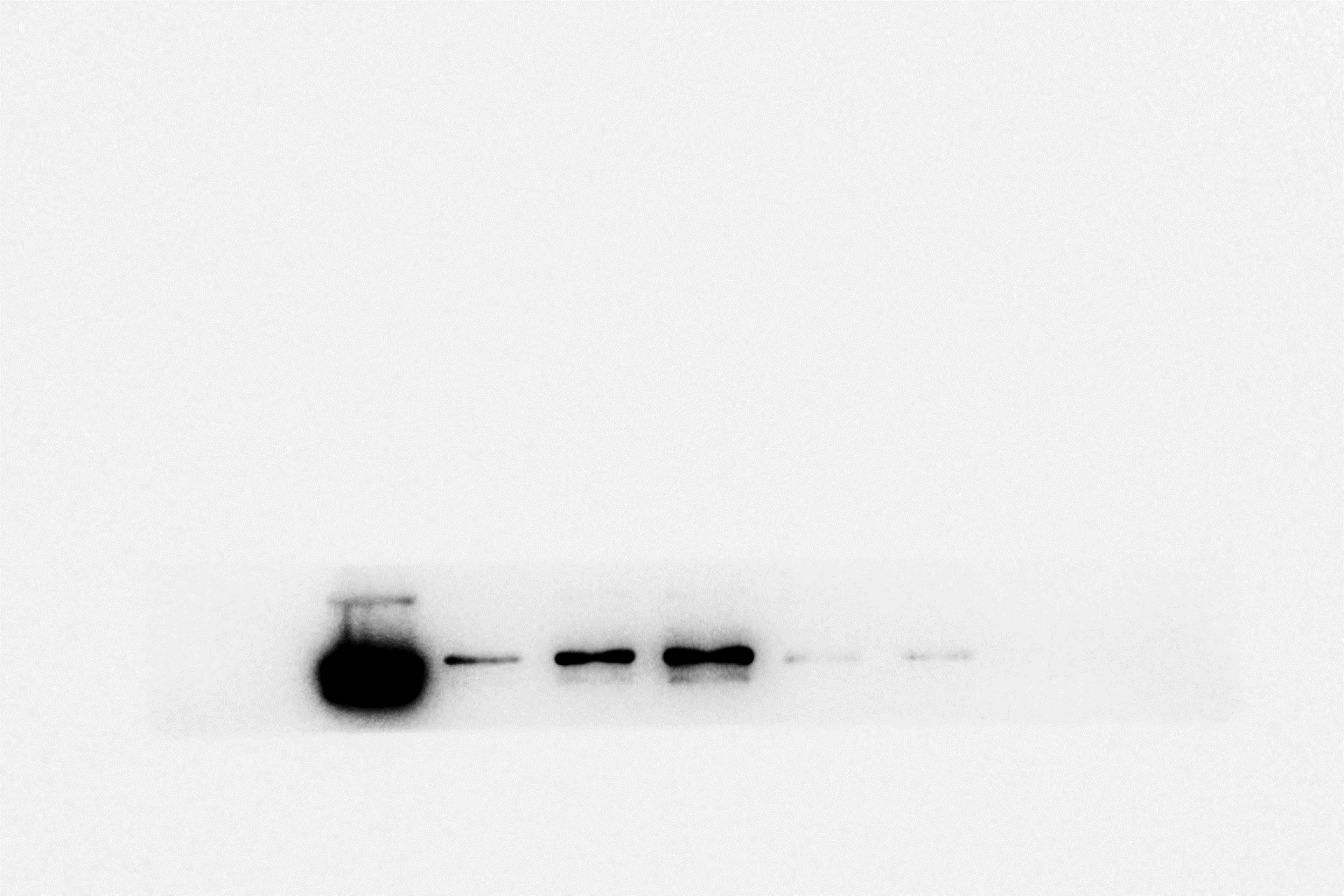

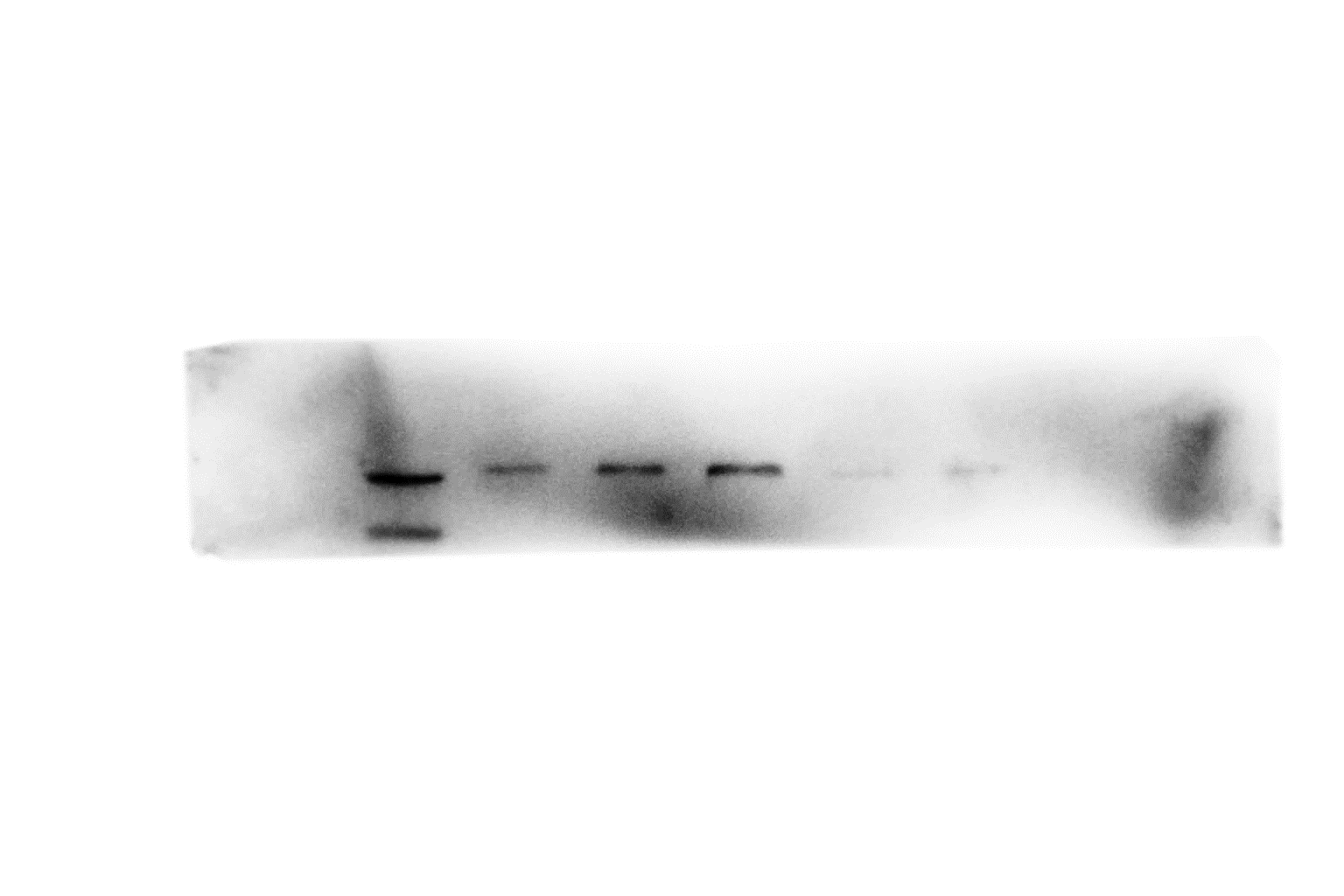


Full length

No RNA

Input

NF90

GAPDH

NF45

35kD

90kD

40kD

P4

P3

P2

P1

i

Figure 5

si-NF90-2

si-NF90-1

si-NF90-2

si-NF90-1

si-NC

si-NC

b









35kD

90kD

40kD

NF90

NF45

GAPDH

input

IgG

IP




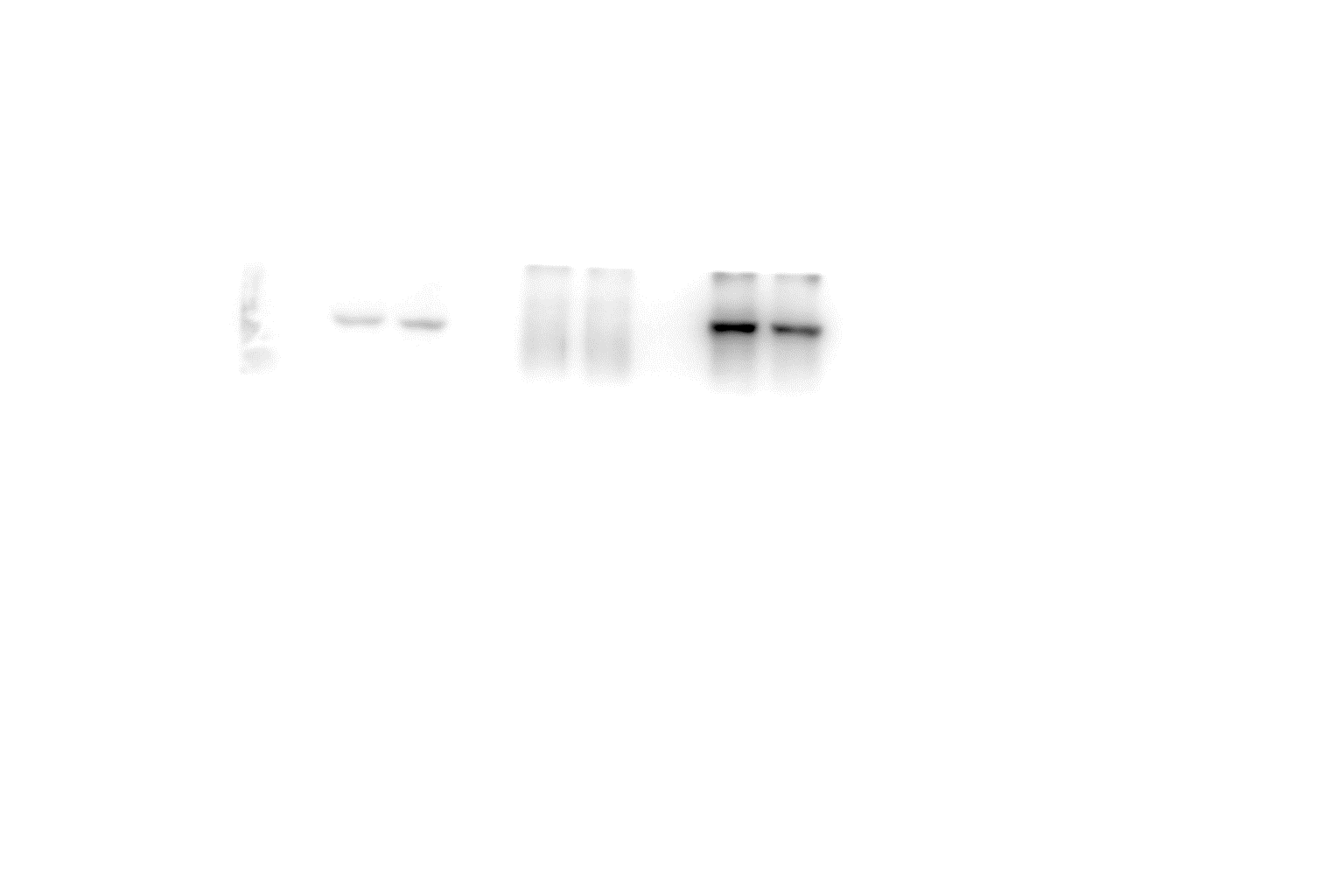



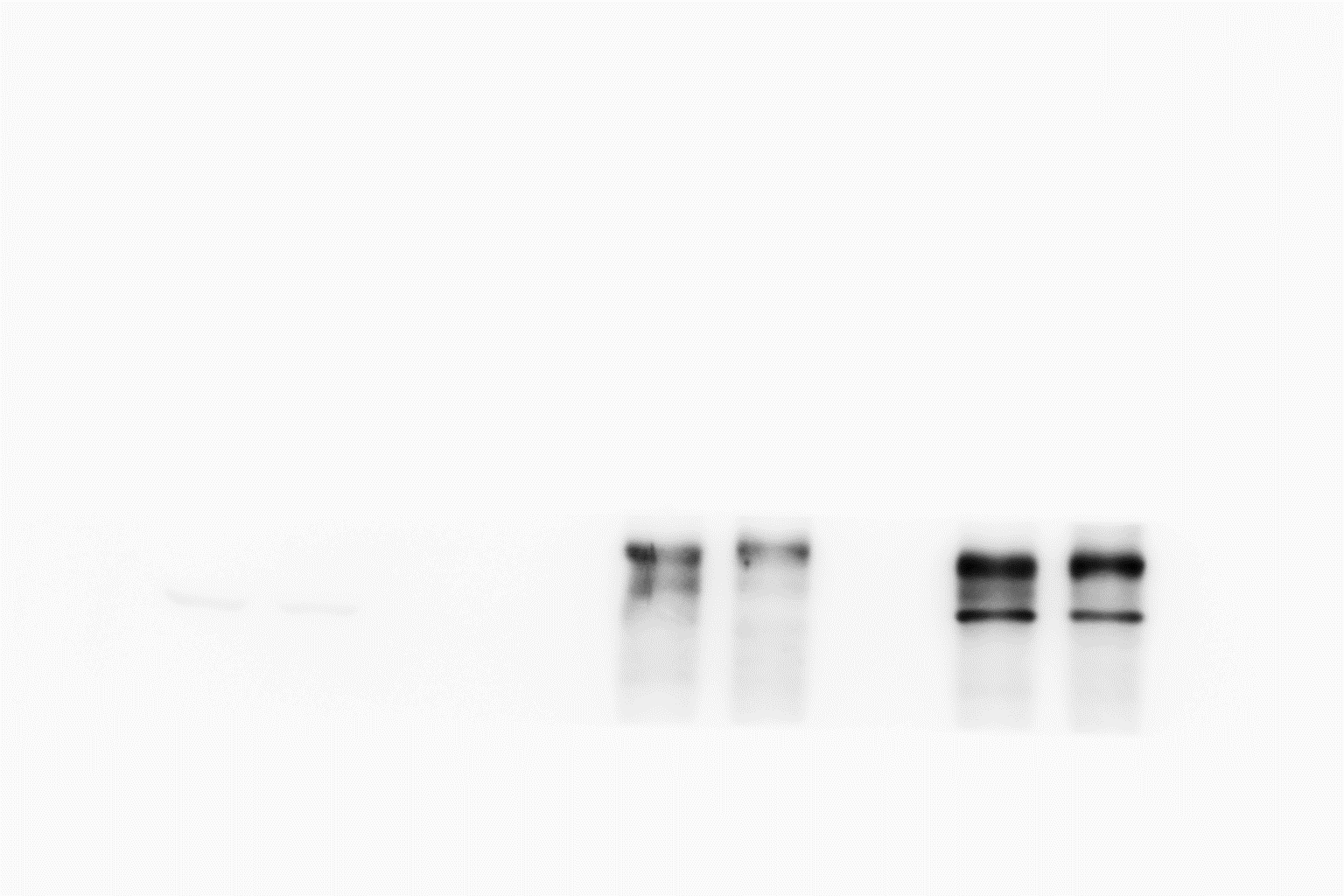


e

90kD

sh-NR038975

40kD

90kD

40kD

NF90

NF90

NF45

NF45

sh-NR038975

sh-NR038975

sh-NC

sh-NC

sh-NC
